# Low-grade myofibroblastic sarcoma of the retroauricular region treated with complete circumferential peripheral and deep margin assessment surgery and local flap construction: A case report

**DOI:** 10.1016/j.jdcr.2026.09.023

**Published:** 2026-09-19

**Authors:** Laura M. Umaña, María Pineda Muñoz, Hector C. Pérez, Javier Hernández, Luis E. Nieto, Christian Acosta

**Affiliations:** a Department of Dermatology, Hospital Militar Central, Bogotá, D.C., Colombia; b Department of Dermatology, Hospital Militar Central and Universidad Nacional, Bogotá, D.C., Colombia; c Department of Pathology, Hospital Militar Central, Bogotá, D.C., Colombia; d Department of Plastic Surgery, Hospital Militar Central, Bogotá, D.C., Colombia; e Department of Plastic Surgery, Hospital Militar Central, Bogotá, D.C., Colombia

**Keywords:** Complete circumferential peripheral and deep margin assessment, dermatology, low-grade myofibroblastic sarcoma, margin-controlled surgery, skin cancer, surgical flaps

Low-grade myofibroblastic sarcoma (LGMS) is a rare malignant mesenchymal tumor characterized by myofibroblastic proliferation. It is classified by the World Health Organization as a fibroblastic and myofibroblastic tumor of soft tissue.^1^ LGMS is commonly found in the head and neck region, presenting as a slowly growing, often asymptomatic mass.^2^ Because of its nonspecific clinical appearance, spindle-cell morphology, and histopathologic overlap with other spindle neoplasms, accurate diagnosis can be challenging. We report a case of LGMS located in the retroauricular region, managed with complete circumferential peripheral and deep margin assessment (CCPDMA) surgery and subsequent local flap reconstruction.

## Case report

A 31-year-old man with no significant medical history presented with a progressively enlarging lesion in the left retroauricular region, first noted approximately 10 months earlier. Physical examination revealed a firm, well-defined nodule that measured 0.5 cm in diameter, with a palpable subcutaneous nodule of 4 cm and a linear scar corresponding to the site of a previous biopsy (Fig 1).

**Fig 1:**
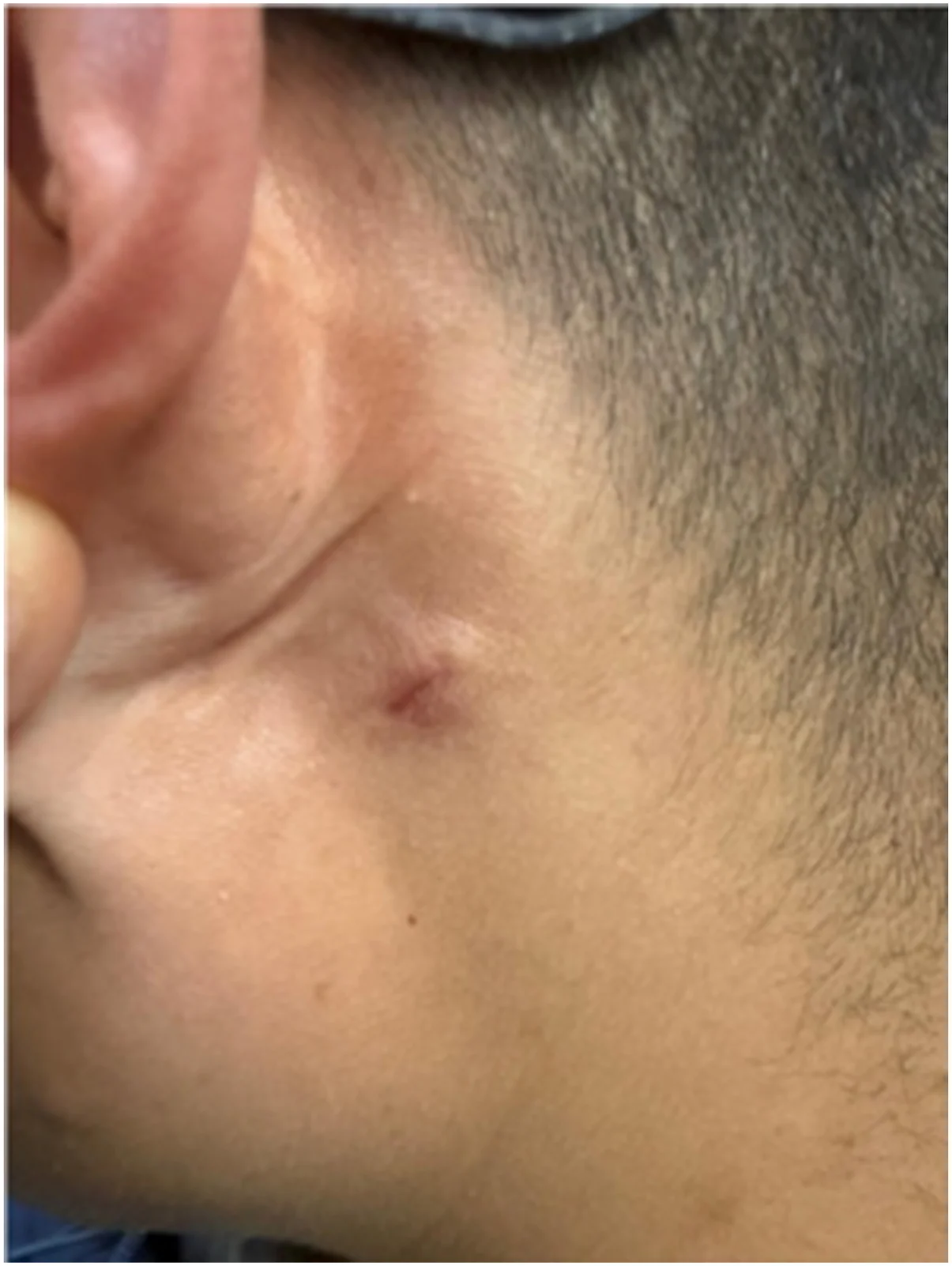
Initial lesion. A firm, well-defined, 0.5 cm in diameter nodule, with a linear scar corresponding to the previous biopsy site.

Histopathologic study revealed a spindle and epithelioid cell malignant neoplasm, scattered in nodular patterns, without necrosis or atypical mitotic figures. Immunohistochemistry showed positivity for smooth muscle actin and h-caldesmon with a Ki-67 cellular proliferation index of 10%, while S100, CD34, desmin, SOX10, CKAE1AE3, HB45, CD45, CK5/6, CD31, ERG, and human herpes virus 8 were negative; findings were consistent with LGMS (Fig 2).

**Fig 2:**
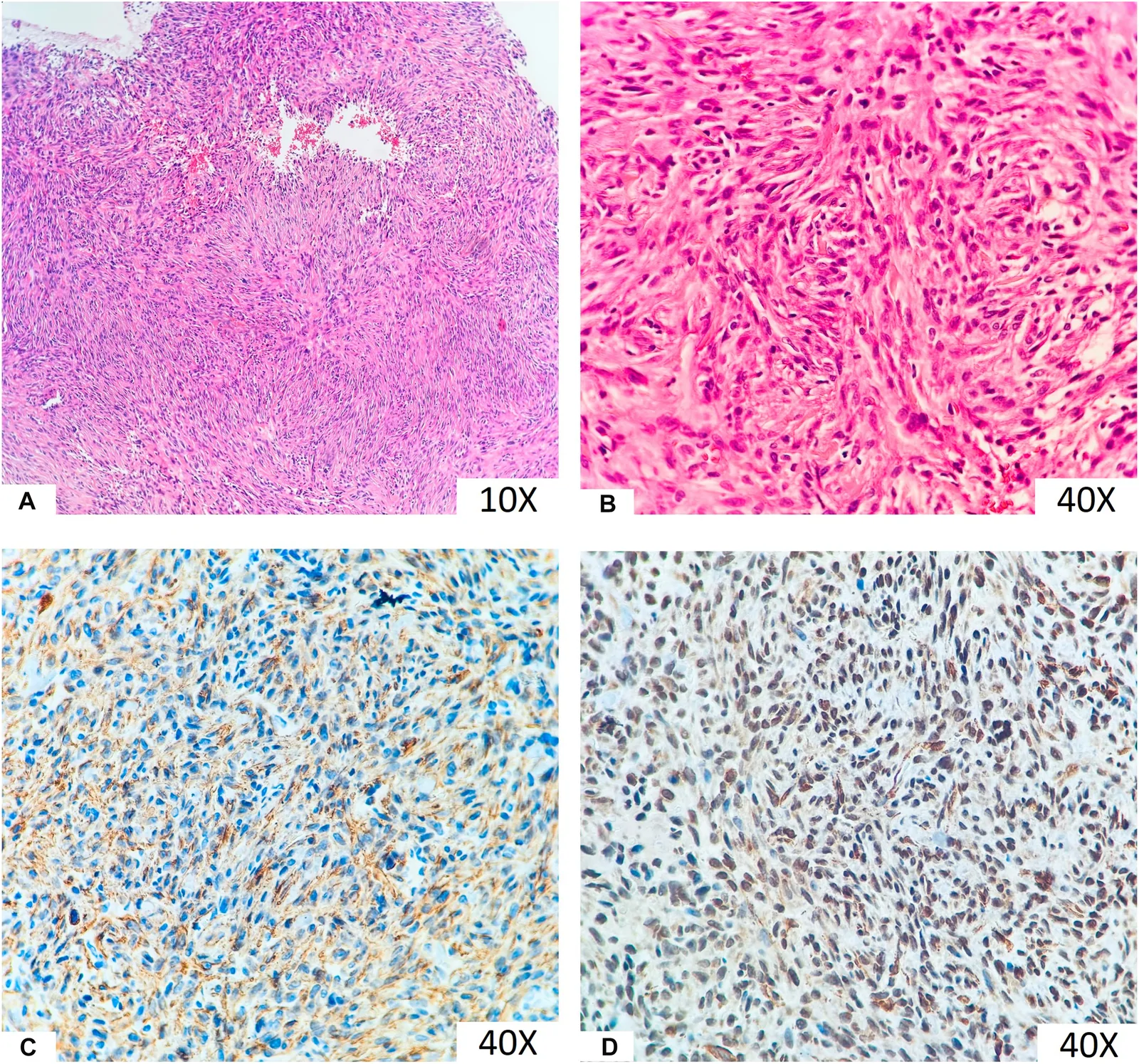
Histopathologic findings of low-grade myofibroblastic sarcoma. **A, B,** Histologic section stained with hematoxylin and eosin showing a highly cellular malignant tumor consisting of proliferating epithelioid and spindle-shaped cells scattered in a nodular pattern. **C,** Immunohistochemical study with diffuse positivity for caldesmon, consistent with myofibroblastic differentiation. **D,** Positive immunostaining for smooth muscle actin (SMA) supporting the mesenchymal origin of the tumor.

Given the known infiltrative behavior and high risk of local recurrence associated with LGMS, the location in the head and neck, and the need to secure negative margins, the case was discussed in a multidisciplinary tumor board. Surgical management with CCPDMA was selected as the preferred therapeutic approach in order to achieve complete margin control while preserving surrounding healthy tissue. Head and neck magnetic resonance imaging with contrast was ordered for presurgical evaluation of subclinical extension and to rule out metastasis because of the rarity of the tumor; no abnormalities were reported in the study.

The surgery was performed with an initial clinical margin of 1 cm. Complete resection of the tumor with histologically negative margins was achieved after 1 stage. The tumor was processed through CCPDMA (Fig 3). The resulting surgical defect of approximately 6 × 6 cm was reconstructed by the plastic surgery department using a local rotation flap (Fig 4), 7 days after the tumor extraction, when the pathologist and surgeon reported negative margins. A fasciocutaneous rotation flap was designed using methylene blue, oriented along relaxed skin tension lines. The procedure was performed under general anesthesia. Incision and dissection were carried out in a layered fashion down to the deep fascia, followed by subfascial dissection, flap elevation, and rotation into the defect. Adequate hemostasis was achieved, and the wound was closed without tension using 5-0 Vicryl sutures for the deep layer and simple and mattress 5-0 Prolene sutures for skin closure. At the patient’s 1 month follow-up, no signs of recurrence were noted. There were no functional or aesthetic complications.

**Fig 3:**
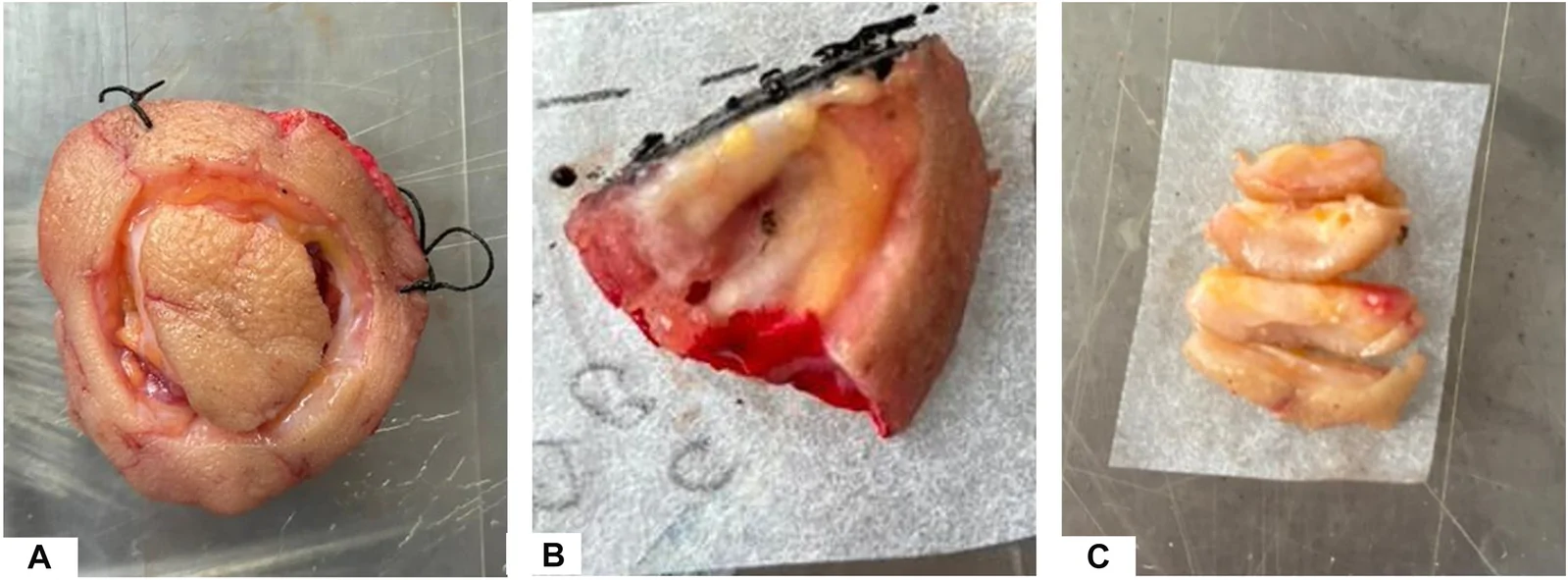
CCPDMA processing of the tumor. **A,** Tumor. **B,** Color coding of the borders. **C,** Debulking of the tumor. *CCPDMA*, Complete circumferential peripheral and deep margin assessment.

**Fig 4:**
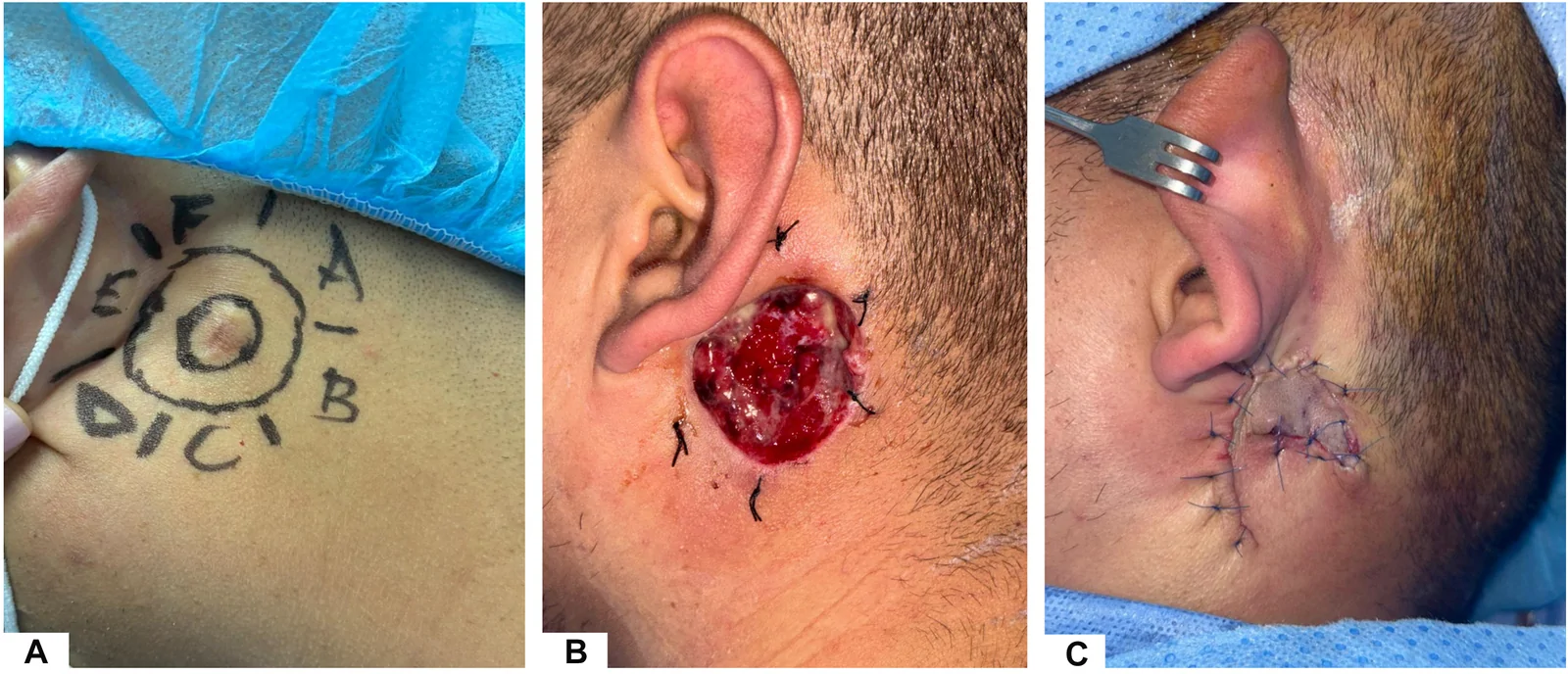
CCPDMA surgery procedure and reconstruction. **A,** CCPDMA surgical marking in the left retroauricular region. **B,** The resulting 6 × 6 cm defect without bone exposure. **C,** Fasciocutaneous rotation flap used for reconstruction of the defect in the left retroauricular region. *CCPDMA*, Complete circumferential peripheral and deep margin assessment.

## Discussion

LGMS is an uncommon soft tissue sarcoma that predominantly affects young middle-aged adults, with a slight male predominance reported in most series.^1^ The annual incidence of this entity is less than 1 per 1,000,000 people.^3^ Clinically, LGMS often presents as a painless, slow-growing lesion, and it shows a marked predilection for the head and neck region.^2^ Histologically, it is characterized by spindle-shaped cells with myofibroblastic differentiation arranged in fascicles, usually showing mild to moderate nuclear atypia and low mitotic activity.^1^ Immunohistochemistry is essential for diagnosis and differential exclusion of other spindle-cell neoplasms. In the present case, the tumor cell showed positivity for caldesmon, consistent with myofibroblastic differentiation, along with positive immunostaining for smooth muscle actin, supporting the mesenchymal origin of the tumor.^4^

LGMS is characterized by an infiltrative growth pattern and a significant tendency for local recurrence of approximately 18.8% following surgery, particularly in cases where excision margins are inadequate.^5^ For this reason, surgical strategies that allow comprehensive margin assessment are of particular importance to avoid recurrence. Previous series have emphasized the need for wide surgical margins in order to reduce the risk of local recurrence in LGMS.^6^ CCPDMA techniques, including Mohs micrographic surgery and paraffin-embedded variants, provide complete circumferential and deep margin control, enabling the identification of subclinical tumor extension that may not be apparent clinically. This approach is advantageous in anatomically important areas such as the head and neck, where tissue preservation is critical.^7^ In the present case, the decision to perform CCPDMA was guided by the known recurrence risk of LGMS, its infiltrative behavior, and the need to preserve functional skin. The tumor was dissected at a 45° angle, and the totality of the borders, as well as the debulking of the tumor, was processed through paraffin given the rarity of the tumor. To our knowledge, there is only 1 known case described in the literature in which CCPDMA surgery including Mohs micrographic surgery was successfully implemented for the treatment of a patient with myofibrosarcoma,^8^ the present case being the second.

Following tumor clearance, reconstruction was performed using a local fasciocutaneous rotation flap. Based on fundamental principles of reconstructive surgery, including the “like-with-like” concept,^9^ the patient was considered an appropriate candidate for local flap reconstruction, allowing effective defect coverage while avoiding the need for regional or distant flaps.

This case highlights the importance of including LGMS in the differential diagnosis of atypical spindle-cell tumors of the skin and supports complete circumferential and deep margin assessment surgery as a valuable tissue-sparing therapeutic approach in anatomically important regions.

### Declaration of generative AI and AI-assisted technologies in the writing process

None.

## Conflicts of interest

None disclosed.
